# Do we need rehabilitation after total knee arthroplasty? Evidence, uncertainty and the need for focus

**DOI:** 10.1002/jeo2.70673

**Published:** 2026-02-27

**Authors:** Thomas Wainwright, Henrik Kehlet

**Affiliations:** ^1^ Orthopaedic Research Institute Bournemouth University Poole UK; ^2^ Physiotherapy Department University Hospitals Dorset NHS Foundation Trust Bournemouth UK; ^3^ Section of Surgical Pathophysiology, Rigshospitalet Copenhagen University Copenhagen Denmark; ^4^ The Centre for Fast‐Track Hip and Knee Replacement Copenhagen Denmark

**Keywords:** physical therapy, prehabilitation, recovery of function, rehabilitation, total knee arthroplasty

## Abstract

The global increase in total knee arthroplasty has led to greater examination of postoperative recovery, particularly the effectiveness and purpose of rehabilitation. Although surgery reliably reduces pain and improves patient‐reported assessments of function, objectively measured physical activity commonly declines after surgery and often remains below preoperative levels for prolonged periods. This mismatch exposes shortcomings in traditional outcome measures and challenges longstanding assumptions about predictable, linear recovery. Emerging evidence from recent trials questions the clinical impact of conventional preoperative and postoperative rehabilitation programmes. At the same time, national registry data show that inadequate restoration of daily activity carries significant long‐term socioeconomic consequences. These findings collectively indicate a need to move beyond protocol‐driven models of rehabilitation towards a deeper understanding of the biological, behavioural and contextual factors that shape recovery. It proposes a conceptual shift towards personalised, dynamically reassessed recovery pathways informed by objective activity measurement, patient‐centred outcomes and mechanistic understandings of postoperative physiology. The central question is not whether rehabilitation is required, but how to identify which individuals need targeted therapy, at what point in their recovery, and through which interventions. Reframing rehabilitation in this way is essential to closing the gap between symptomatic improvement and meaningful functional recovery after knee replacement surgery.

**Level of Evidence** N/A, narrative commentary.

## Graphical abstract

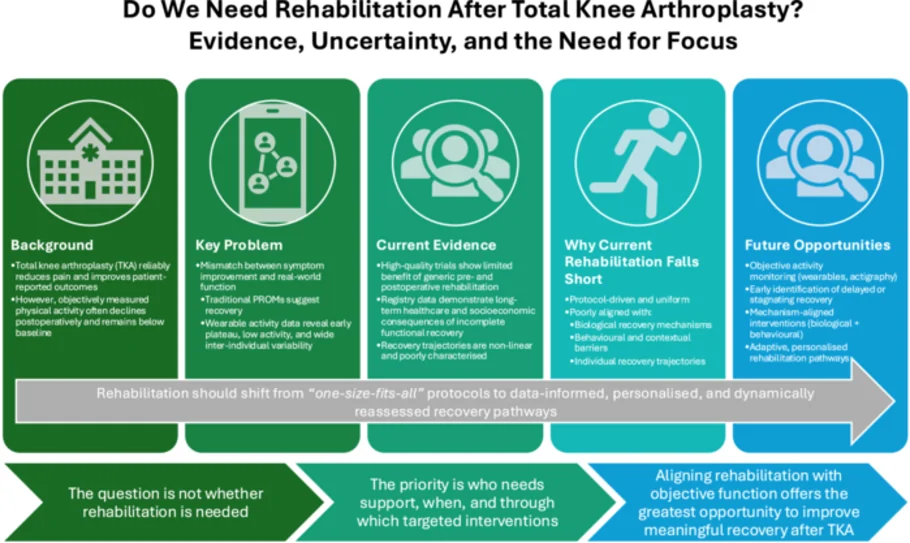

**Abbreviations**
BMI: body mass index
PROM: patient reported outcome measure
RCT: randomised clinical trial
THA: total hip arthroplasty
TKA: total knee arthroplasty

The global incidence of total knee arthroplasty (TKA) continues to rise annually. Although TKA is now associated with high technical reliability, improved implants and streamlined surgical pathways, uncertainty remains regarding optimal rehabilitation. Overall, patients report reduced pain and improved function following surgery. However, objective measures indicate that physical activity levels after TKA remain low compared to preoperative values and population norms. Recent studies employing actigraphy and accelerometers have identified a paradox: patients experience symptom improvement but remain physically less active during early recovery [11, 14, 17]. For decades, rehabilitation interventions, including prehabilitation and postoperative rehabilitation, have been presumed to address this gap. Emerging evidence now challenges the effectiveness of these approaches and prompts a re‐evaluation of rehabilitation needs following TKA.

Traditional outcome measures, particularly patient reported outcome measures (PROMs), indicate strong functional recovery after TKA. However, objective activity tracking provides a contrasting perspective. Studies using step counts and active time from devices such as Fitbit or medical‐grade actigraphy demonstrate that patients' activity levels decline during the first 6 weeks postoperatively and often remain below baseline for an extended period [11, 14, 16]. Although patients with higher preoperative function or physical activity levels tend to recover more rapidly, the correlation is less robust than clinicians commonly assume [13, 14, 15]. Other potential predictors, including age, sex, body mass index (BMI) and psychological readiness, exhibit inconsistent or weak associations with early mobility [4, 15]. While PROMs suggest continuous improvement in function after surgery, activity data reveal an early plateau and substantial interindividual variability. This discrepancy challenges the prevailing assumption of a steady, linear recovery trajectory [17]. These findings indicate that future progress in rehabilitation after TKA is unlikely to come from further refinement of generic protocols, but rather from approaches that better characterise recovery trajectories, identify modifiable barriers to activity and target interventions accordingly.

These challenges are mirrored in the wider perioperative literature. The recent international consensus process by El‐Boghdadly et al. [3] highlights that outcome selection in perioperative and regional anaesthesia research remains highly inconsistent and insufficiently patient‐centred, arguing for more multidimensional and functionally relevant evaluation frameworks. Their prioritised outcomes explicitly emphasise functional recovery, cognitive recovery and quality‐of‐recovery domains, none of which are adequately captured by traditional PROMs alone. Adoption of such frameworks strengthens the case for integrating objective activity monitoring into arthroplasty research and routine care.

The lack of increased physical activity despite symptom improvement necessitates a critical evaluation of the effectiveness of current rehabilitation protocols in promoting physical activity. The implications of persistently low physical activity extend beyond clinical recovery. A nationwide Danish register study including over 120,000 TKA and total hip arthroplasty (THA) patients demonstrated that, despite technically successful surgery and access to conventional rehabilitation, patients consistently required greater healthcare utilisation and support services than matched controls for many years after surgery [10]. Increased outpatient care, higher prescription medication use, greater reliance on home‐care services and sustained reductions in employment income were observed. These data highlight that inadequate restoration of functional capacity and daily activity carries substantial long‐term socioeconomic consequences, stressing the need to rethink whether current rehabilitation models effectively promote meaningful recovery.

Recent high‐quality trials have questioned the effectiveness of current and accepted rehabilitation. The DRAW1 trial, a large pragmatic randomised controlled trial (RCT), compared structured rehabilitation, including tele‐rehabilitation and home‐based care, to no rehabilitation following TKA and THA [12]. The trial found no significant differences in function, pain or performance outcomes at any measured time point [12]. Similarly, Bandholm et al. contend that prehabilitation lacks sufficient evidence to support its routine implementation, particularly when based on assumptions rather than empirical data [1]. However, these findings do not render rehabilitation obsolete. Instead, they necessitate a reassessment of which patients benefit from rehabilitation, the optimal timing and the underlying rationale. They should be interpreted not as evidence against rehabilitation per se, but as an opportunity to redesign rehabilitation around patient selection, timing and mechanism‐specific targets rather than uniform programme delivery. Future trials should therefore prioritise stratified or adaptive designs, incorporating objective activity monitoring to identify early divergence in recovery trajectories and to trigger targeted, time‐sensitive interventions.

A more productive focus for future research may be to develop a deeper understanding of recovery following TKA, rather than solely evaluating the efficacy of specific rehabilitation protocols. The pathophysiology of postoperative recovery remains inadequately characterised [8, 17], and future trials will expand more on whether characterisation is possible, and the details of recovery trajectories [6]. Although enhanced recovery programs have reduced surgical stress and shortened hospital stays, these improvements have not consistently resulted in faster or superior functional recovery. In the absence of a comprehensive biological, behavioural or contextual model for regaining real‐world activity, clinical practice often relies on tradition rather than evidence. Patients may remain inactive despite being pain‐free due to factors such as habit, fear, insufficient guidance, minor procedure‐related complications or environmental barriers. Most PROMs may be unable to capture these nuanced influences, and current activity data indicate that rehabilitation programs frequently do not address them. This is consistent with recent critiques of perioperative trial design, which describe how unimodal interventions, whether analgesic, surgical or rehabilitative, are unlikely to influence recovery unless framed within a comprehensive understanding of postoperative pathophysiology. Kehlet and Lobo [9] emphasise that effective interventions must correspond to the duration and biological mechanisms of postoperative inflammatory, endocrine and neuromuscular responses. Applied to TKA rehabilitation, this suggests that short‐duration, protocolised or generic rehabilitation programmes may be misaligned with the biological determinants of recovery, explaining the limited clinical impact demonstrated in recent trials.

A paradigm shift is therefore required, moving from generalised, protocol‐driven rehabilitation towards personalised, behaviourally informed care pathways. In the postoperative context, wearable actigraphy is already identifying previously unrecognised recovery trajectories. As an example, a study of functional recovery after thoracoscopic lung cancer lobectomy in a well‐established enhanced recovery program [5], with a short length of stay (3 days), activity measurement showed a 40% decline in the first week after discharge. The study added a questionnaire on reasons for reduced activity (PROM), clearly showing that two factors were dominant, namely pain and fatigue, thereby serving to suggest that future interventions to improve functional recovery by reducing the inflammatory response (by glucocorticoids) [7] or through improvements to multimodal analgesic regimes.

These technologies present a clear opportunity to shift rehabilitation from a retrospective, protocol‐driven model towards a prospective, data‐informed approach, in which rehabilitation intensity, content and duration are dynamically adjusted based on objectively measured recovery. Recovery pathways could be adapted based on step counts, early identification of stagnation, and the application of behaviour‐change models to promote increased movement. Rather than abandoning rehabilitation, it is necessary to determine which patients benefit from structured rehabilitation, the optimal timing for intervention, the most effective types of rehabilitation and the appropriate duration. Technology should be leveraged to individualise and adapt care. To achieve this, structured, repeated assessments of rehabilitation needs must be conducted both before surgery and at key postoperative milestones, ensuring that rehabilitation decisions are informed by evolving clinical risk, functional status and patient goals [2]. The central question is not whether rehabilitation is needed, but whether the appropriate support is being provided to the right patients at the right time.

Future rehabilitation trials must therefore move beyond comparing generic rehabilitation programmes against minimal care. Instead, designs should integrate continuous objective activity monitoring, identify early stagnation or divergent trajectories and deploy personalised, mechanism‐aligned interventions targeting the biological and behavioural determinants of recovery. Such designs would adhere to contemporary recommendations for procedure‐ and patient‐specific outcome frameworks and are more likely to yield clinically meaningful improvements in postoperative mobility.

In summary, the observed disconnect between symptom improvement and physical activity following TKA requires immediate attention. Rehabilitation should be refined rather than defined per tradition. Clinical practice must move beyond assumptions, reliance on PROMs and traditional approaches, adopting a new understanding of recovery that incorporates behaviour, context and objective functional measures. Future progress in TKA rehabilitation will depend on abandoning assumptions of uniform recovery and embracing personalised, adaptive models that integrate biological mechanisms, behavioural drivers and objective functional measurement. Such an approach offers the opportunity not only to improve individual patient results but also to reduce long‐term healthcare utilisation and societal burden associated with incomplete functional recovery.

## AUTHOR CONTRIBUTIONS

Thomas Wainwright and Henrik Kehlet conceived the manuscript. Thomas Wainwright drafted the initial manuscript with revision and input from Henrik Kehlet. Both authors had final responsibility for the decision to submit the manuscript for publication.

## CONFLICT OF INTEREST STATEMENT

Thomas Wainwright reports nonrelated institutional research funding from the National Institute of Health and Care Research (NIHR), Stryker and Zimmer Biomet; and personal fees from Pharmacosmos, Firstkind, Enhanced Medical Nutrition and Molnlycke Health Care.

## ETHICS STATEMENT

The authors have nothing to report.
